# Improvement in Domestic Violence and Aggression in Comorbid ADHD and Conduct Disorder Following Methylphenidate Initiation: A Case Report

**DOI:** 10.7759/cureus.115810

**Published:** 2026-09-05

**Authors:** Zaid Mamser, Victoria Norrington-Moore

**Affiliations:** 1 Psychiatry, Lancashire and South Cumbria NHS Foundation Trust, Preston, GBR; 2 Child and Adolescent Psychiatry, Forensic Psychiatry, Children and Young People’s Mental Health Services East, Lancashire and South Cumbria NHS Foundation Trust, Burnley, GBR

**Keywords:** attention-deficit hyperactivity disorder (adhd), child and adolescent psychiatry, domestic abuse, psychiatry and mental health, stimulant treatment

## Abstract

Attention-deficit hyperactivity disorder (ADHD) and conduct disorder (CD) are diagnoses that are frequently comorbid. CD is characterised by persistent dissocial, aggressive, and defiant behaviors that defy age-appropriate expectations.

This is the case of a 15-year-old male adoptee referred to Tier 3 Child and Adolescent Mental Health Service (CAMHS) for severe aggression, impulsivity, and domestic violence. The patient exhibited coercive control toward his mother, physical violence involving weapons, and property damage, necessitating police involvement and risking family placement breakdown.

Following a failure of psychosocial interventions, the patient underwent the Quantified Behavioral (Qb) Test and Conners-4 assessments and review by a consultant psychiatrist confirming ADHD and conduct disorder diagnosis. Methylphenidate was initiated and titrated to 36mg daily.

Outcomes were significant. Within one month, antisocial behaviors reduced markedly. At seven months, domestic violence had ceased, and social care involvement ended. The patient successfully completed his General Certificates of Secondary Education (GCSE). Repeat testing on medication showed normalisation of scores: QbTest activity dropped to the 23rd percentile and impulsivity to the 21st percentile. Crucially, parent-rated Conners-4 scores for CD dropped to zero.

This case highlights the importance of identifying and treating underlying ADHD in adolescents presenting with severe conduct issues. Higher doses of methylphenidate are often cited as beneficial for CD symptom reduction; this patient achieved remission of aggression at 36mg/day. The case shows that delaying ADHD diagnosis in favor of solely managing behavioral conduct symptoms can risk education and family stability. Treating the ADHD effectively mitigated the emotional dysregulation and violence and reduced the negative impact on this patient during a crucial period of his life as he was completing high school.

This case highlights the importance of considering comorbid ADHD and CD in patients presenting with impulsive behaviours and aggression as their primary presenting complaints. Appropriate management with methylphenidate could have a significant therapeutic effect on these patients and their antisocial behaviours.

## Introduction

Attention-deficit/hyperactivity disorder (ADHD) is a neurodevelopmental disorder characterised by developmentally inappropriate inattention, hyperactivity and impulsivity. The International Classification of Diseases, 10th Revision (ICD-10) designates it as a hyperkinetic disorder, describing early onset together with an inability to persist at tasks demanding cognitive effort, abandonment of activities before completion, and motor activity that is both excessive and poorly organised. Affected children are further characterised as impulsive and accident-prone, and as incurring disciplinary sanction through thoughtlessness rather than intent [1]. The Diagnostic and Statistical Manual of Mental Disorders, Fifth Edition, Text Revision (DSM-5-TR) requires several symptoms to be present before the age of 12 years, across two or more settings, with clear evidence of functional impairment [2]. Population surveys place worldwide prevalence in children at approximately 7.2%, although cross-national estimates range widely; heritability is approximately 74%, with no single causative gene identified. Rates are higher in populations marked by early adversity, notably children in foster care and young people in correctional settings [2], a point of direct relevance to the patient described here, who entered care in infancy.

Conduct disorder (CD) is defined in ICD-10 as a repetitive and persistent pattern of dissocial, aggressive or defiant behaviour that exceeds age-appropriate social expectations. Cited examples include excessive fighting or bullying, cruelty to people or animals, severe destructiveness, fire-setting, stealing, truancy and repeated severe temper outbursts. An isolated act is insufficient; the diagnosis requires that such behaviour be pervasive and enduring [1]. DSM-5-TR reports a one-year population prevalence of between 2% and more than 10% across the United States and comparable high-income countries, with a median of 4%, and a lifetime prevalence in the United States of 12.0% among men and 7.1% among women [2]. Its recognised risk factors overlap substantially with those for ADHD and cluster within the caregiving environment: rejection, neglect and inconsistent or punitive parenting, maltreatment, institutional care in early life and repeated changes of caregiver, alongside parental criminality and psychiatric illness [2]. This constellation is frequently encountered among children adopted after a period in care.

The two disorders co-occur frequently. DSM-5-TR reports CD in approximately one quarter of children and adolescents with ADHD of combined presentation [2], and a large population-based study found comorbid CD in 27% of individuals with ADHD [3]. ICD-10 treats the overlap as a discrete entity, hyperkinetic conduct disorder (F90.1), which is to be coded in preference to two separate diagnoses when criteria for both are satisfied [1]. The comorbidity matters clinically because it carries greater aggression, poorer educational attainment and higher risk of justice involvement than either disorder alone. It also creates a diagnostic hazard: where aggression and violence dominate the presentation, they may be attributed wholly to CD, and an underlying and treatable ADHD may be overlooked or its assessment deferred.

Management differs accordingly. For CD, the National Institute for Health and Care Excellence (NICE) recommends psychosocial intervention as the mainstay parent training programmes, child-focused problem-solving programmes and, for young people aged 11 to 17 years, multimodal family-based interventions delivered by specifically trained case managers and advises against routine pharmacological treatment, reserving risperidone for short-term management of severe aggression in those who have not responded to psychological approaches. Where ADHD is comorbid, however, methylphenidate or atomoxetine is recommended [4]. For ADHD in children aged over five years, NICE advises psychoeducation and environmental modification in the first instance, with medication offered where symptoms continue to cause significant impairment in at least one domain; methylphenidate is first line, lisdexamfetamine second line, and atomoxetine or guanfacine are reserved for those who cannot tolerate or do not respond to stimulants [5]. The evidence for stimulant treatment in comorbid ADHD and CD is encouraging but limited, and it has been suggested that comparatively high doses may be required before conduct symptoms improve [6-10].

The following report has been written about a 15-year-old Caucasian male, adopted with his younger biological brother at two years and 11 months of age, who was referred to a Tier 3 (specialist) Child and Adolescent Mental Health Service in the United Kingdom with severe aggression, impulsivity and domestic violence. His difficulties had not responded to psychosocial intervention, had prompted repeated police attendance and statutory social care involvement, and had brought his adoptive placement close to breakdown during his final year of secondary education. The aim of this report is to describe the diagnostic process by which comorbid ADHD was identified in a presentation dominated by conduct symptoms, and to document the clinical, educational and family outcomes that followed initiation of methylphenidate.

## Case presentation

Presenting complaint

The patient presented with significant challenging behaviour, though noted starting from the age of four, with comments such as ' will stab you' as well as kicking his adoptive mother. At 11 years old, these behaviours became severe enough to prompt assessment by a clinical psychologist at the request of their post-adoption social workers, after which he was offered and completed CBT and therapeutic parenting sessions in 2022; his parents only noted a slight improvement in his behaviour after these interventions. He was described by his mother as ‘going from 0 - 100 in nanoseconds’ and then calmed down relatively quickly. It was noted that there was near-daily violence at home, with police visits as a result. Parents noted that when boundaries were put in place, e.g., removing his phone from him, the patient would escalate his behaviour and refuse to comply. He would try to provoke arguments and then retaliate in a violent manner. Instances of violence include verbal abuse, physical abuse such as punching, kicking, or dragging his mother by the hair, and the use of weapons such as knives, directed at his parents as well as his brother (who is 10 months younger). 

His parents had to lock themselves in their bedroom to protect themselves from this violence. Despite police visits, the patient had not been arrested or cautioned. He primarily subjected his mother to coercive control and intimidation. The patient would present himself ‘as the victim’ and not take responsibility for his behaviour. 

The patient was noted to be violent in the community. This included when he was 11 years old in year 7 of school, hitting a friend in the face with a plank of wood after an argument. The patient had been involved in stealing money from home. He had been involved in posting inappropriate posts on social media, putting himself in positions where he was vulnerable to sexual exploitation. He impulsively attempted to purchase a dog online as well as order a stairlift and life insurance for his parents. 

The patient described his mood as being low but was unable to identify any precipitating factors. He described anhedonia, as he felt no pleasure in his day-to-day activities and hobbies. He has difficulties with concentrating on any tasks and becomes easily frustrated. He described only being able to focus on social media and would move from one interest to another. The patient described poor sleep, staying up significantly past midnight, and having poor sleep hygiene, e.g., watching television late at night. His mother had noted that he would ‘rock himself to sleep at night ’, which was a long-standing issue. His appetite was normal. He stated that he felt anxious. The patient described that he would deliberately provoke others to get a reaction. 

During periods when his mood is calm and settled, he was described to be friendly and pleasant in interactions with both his adoptive parents and his biological brother, though these periods had become fewer over the last few years.

Drugs and alcohol

He had no history of any drug or alcohol misuse. He started to use e-cigarettes daily from 11 years old to the present.

Social history

The patient previously participated in a drama group and enjoyed music and acting, but he stopped attending following interpersonal conflicts. He continues to experience significant difficulty forming and maintaining friendships. While school staff observe close relationships with several peers, his mother notes that these peers also have additional needs. Unlike his brother, who maintains friendships both in and out of school, the patient remains socially isolated and has resorted to communicating with unknown individuals online. Concerns were noted regarding his emerging psychosexual development and online safety. In February 2025, after confusion regarding his sexuality was raised, he attempted to covertly bring an older boy into the family home; this was discovered when his mother found the boy hiding in the garden. Additionally, his Snapchat account was permanently terminated for "abusive behaviour”. Following an argument with a girlfriend, he instructed her to commit suicide and subsequently recorded video footage of her self-harming.

Family history

It has been reported that the patient’s biological parents had significant mental health difficulties. His birth mother was allegedly using cannabis during her pregnancy. His birth father was reported to have a diagnosis of emotionally unstable personality disorder, depression, and anxiety. It was also reported that his maternal birth grandfather had a diagnosis of schizophrenia and his maternal birth grandmother had a diagnosis of depression.

Due to the recent escalation in violence, his father had to seek help from his General Practitioner. The patient was adopted with his biological brother, who was 10 months younger than him. Although his brother presents with similar challenges to the patient, he is more amenable.

Developmental history

The patient’s birth mother struggled during pregnancy, experiencing domestic abuse (including self-harm/threats to harm the patient) and using cannabis. Following birth via emergency C-section due to the umbilical cord around his neck, the patient was placed on an Emergency Protection Order and moved to a foster carer one week later.

The infant reportedly settled well and socialized but had feeding/digestive issues. He initially had contact with birth parents five times weekly. He moved to a second foster placement at five months old, settling well. His birth mother struggled to bond with the patient. Contact with the birth father required two supervisors due to his unpredictability and was suspended after he was aggressive toward the birth mother. Her contact became intermittent and then stopped. The patient’s younger brother was born when the patient was 10 months old. The brothers were initially fostered separately but were placed together 10 months later. Following assessments, it was recommended that both children be adopted due to the birth father’s inability to provide long-term appropriate care.

The adoptive mother noted the patient had delayed developmental milestones (slow to walk and talk). Early in the placement, he was less demanding than his brother but was occasionally aggressive toward him. In early years, he received individual and group speech and language therapy, noting some improvements.

Educational history

At presentation, the patient was in his final year of General Certificate of Secondary Education (GCSE) in Year 11 of school; he attended a mainstream primary school. He was diagnosed with a mild/borderline learning disability post-adoption and was assessed as having sensory issues. He subsequently obtained an EHCP. The patient moved to a mainstream secondary school with support. He socialised with peers who have additional needs but experiences interpersonal difficulties, such as misunderstanding of social cues and difficulties with emotional regulation, leading to incidents with his peers. In school, he was noted to be controlling; sanctions caused disruption as he immediately demanded to know the punishment, timing, and reason. Despite predicted GCSE grades of 1, the patient anticipated that he would obtain grade 5s with the intention of joining the police.

Forensic history

Despite approximately four police attendances during which the patient was aggressive and threatening toward officers, he has no convictions. His violence toward his parents and younger brother includes physical assaults (punching, pushing, dragging, and grabbing his mother by the neck/clothing), weapon use (threatening family with knives), throwing objects (e.g., a gaming chair), and property damage ("smashing up the house", destroying his mother's mobile phone, and emptying her handbag). These behaviours escalate when immediate demands are unmet or sanctions (e.g., removing electronics) are imposed. His parents installed a bedroom door lock to retreat to during escalations, as attempting to disarm him increased their risk of harm. He displays a pattern of presenting as remorseful and distressed to police before resuming violence immediately after they leave, and he has made false complaints against his family.

The patient lacks insight into his conduct, justifying assaulting his mother because "she deserved it", stealing £100 from his aunt, and posting fight videos and provocative conversations on social media. Police were also involved regarding his possession of indecent images. Additional history includes fire-setting three years prior (candles and a hot iron on the carpet) and joining an LGBT group at age 14 despite identifying as heterosexual, which caused distress when he was linked to being gay alongside homophobic bullying from his brother. No animal cruelty or other sexualised behaviours were reported beyond online risk-taking.

Mental status examination

The patient was alert; he was appropriately dressed and had good self-care. He had abnormal eye contact at times staring. No abnormal movements observed. He was fidgety with his hands. He cooperated with the assessment but was quite hostile and argumentative with his mother if he did not agree with what she was saying. He could become more excitable when talking about certain topics such as the police. He had a normal rate, flow, and form of speech. He described his mood subjectively as low most of the time; objectively his mood appeared euthymic with normal variation of affect. He had no thought interference but said he sometimes thinks that something external is controlling him but was unable to expand. He describes some social anxiety and some paranoid ideas about people looking at him and judging him. There were no clear delusional beliefs. He said that he sometimes hears voices when he is trying to get to sleep, both internal and external, but unable to describe the details. No visual hallucinations. Describes having hypersensitivity to smells. No current thoughts to harm self or others. During our assessment, cognition was not tested, though he presents as more able than what his historical cognitive testing indicates. He lacked insight into his difficulties and the impact his behaviour has on others.

Impression

It was felt that the patient had evidence of ADHD due to his issues at school around difficulty sustaining attention, fidgeting, not being able to focus when spoken to, difficulty organising his tasks, and distractibility both at home and at school. These symptoms were assessed against the DSM-V TR criteria [2] along with the data described above from the QB and Conners 4 tests. Additionally diagnosed with CD due to his propensity to engage in physical fights, use of a weapon, destruction of property, fire setting, and theft of money. This was also assessed against the DSM-V TR criteria [2], along with Conners 4, which has a section on CD symptoms. Previously, autism spectrum disorder (ASD) had been suggested, and an assessment was being sought, as ASD is assessed and managed by Neurodevelopmental Pediatrics in this organisation, a referral was made. Psychosis was excluded as a diagnosis. 

ADHD assessment

The patient was reviewed four months later after completion of ADHD assessments completed by the team ADHD practitioner, the QB test [11] showing raised scores in activity 3.3, 99th percentile, attention 2.0, 98th percentile and impulsivity 3.4, 99th percentile. The patient, parents, and school completed the Conners 4 questionnaire [12], which showed raised scores, particularly for inattention and impulsivity. Developmental history suggested longstanding difficulties with attention, concentration and impulsivity. Therefore met the criteria for a diagnosis of ADHD, combined type, and unsocialised CD were made primarily using the DSM-5TR criteria and assessment by a dual trained child and adolescent and forensic psychiatry consultant. At this point the patient was still engaging in antisocial behaviours including suspensions at school, sharing an inappropriate video of his ex-girlfriend as well as aggressively confronting a peer at school. He had involvement from both the Child and Youth Justice Service (CYJS) diversion team who provide mentoring and education/training support and the Children’s Social Care adolescent support unit, who assisted in arranging respite placement for child away from the family home to prevent breakdown of his adoption. Unfortunately these interventions did not improve his behaviour or family situation adequately. The decision was made to initiate methylphenidate modified release, starting at 18mg once a day for seven days, then increased to 27mg once a day for seven days, and finally 36mg once a day from then onwards. No other psychological therapy was offered during this period. Figure 1 shows a timeline of events with the patient's progression.

**Figure 1:**
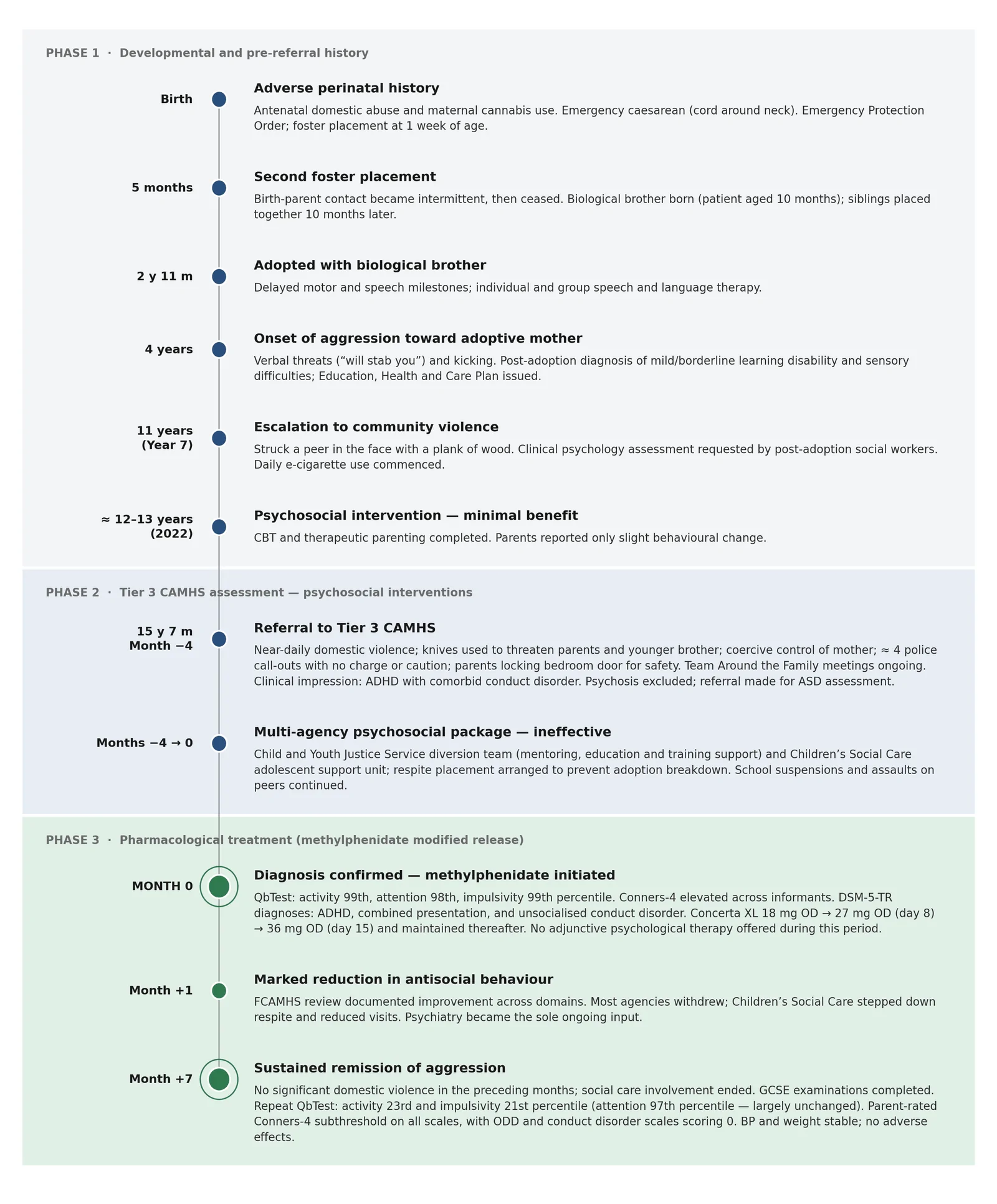
Clinical Timeline Figure created using Matplotlib version 3.10.8 for Python version 3.12.3

His blood pressure and weight were assessed and followed up while on methylphenidate with no abnormalities or concerns noted and no side effects or adverse effects were noted on methylphenidate.

One month after initiation of methylphenidate, the Forensic Child and Adolescent Mental Health Services (FCAMHS) review was completed, and it was noted that the patient had improved in his presentation with regards to antisocial behaviours. With most services ending their inputs, and social services stepping down their involvement in arranging respite and reducing visits. At this point it was only us in the psychiatric service who had regular input into the patient's care.

At a further psychiatrist review seven months post initiation of methylphenidate, and it was noted that the patient had significantly improved and was able to complete his final year of GCSEs. His mother also noted that this aggression was significantly improved since starting on methylphenidate with no significant violence at home in the preceding months. 

A repeat QB test [11] was done while on methylphenidate which showed improvement, activity -0.7, 23rd percentile, attention 1.9, 97th percentile and Impulsivity -0.8, 21st percentile, as shown in Figure 2. Parent Conners 4 questionnaire was also repeated showing all sections to be subthreshold. Notably scoring for both oppositional deficient disorder and CD had dropped to 0 in the repeat questionnaire, supporting the improvements reported at clinic appointments such as improvement in participation at school and ability to complete his GCSEs, a noted improvement in his interpersonal relationships with his family members as well as a cessation of violent behaviours at home and at school. Though some issues around concentration and attention persist though improved.

**Figure 2:**
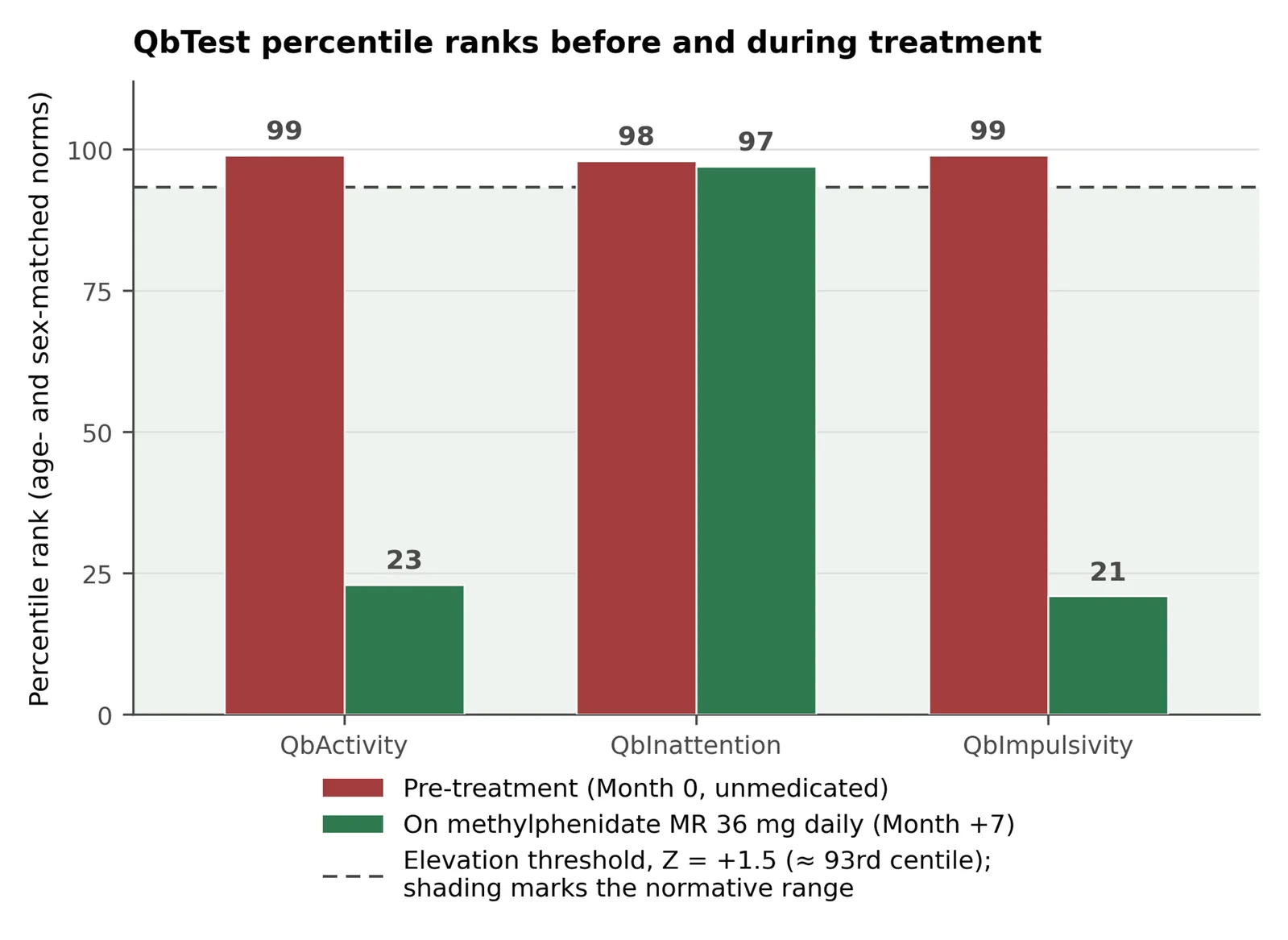
Qb Test Chart Figure created using Matplotlib version 3.10.8 for Python version 3.12.3

Informed consent was obtained from the patient's guardian with parental responsibility with regard to this case report.

## Discussion

This case highlights the significant impact that appropriate diagnosis and management of ADHD can have on a patient's outcomes with regard to the reduction of antisocial behaviour and improvement in family life. While methylphenidate is a first-line treatment in ADHD with a significant amount of evidence to support its use in this case [6], in CD, the mainstay of treatment is psychological therapies, which in this case the parent and child had undergone without success. The NICE guidelines do suggest the use of methylphenidate in cases where CD is co-morbid with ADHD [6]. A case report on a similar case described escalation of antisocial behaviour after methylphenidate discontinuation [13].

There have been studies that support the use of methylphenidate in patients with comorbid ADHD and CD [7-9]. There is a significant short-term decrease in antisocial and aggressive behaviours as well as impulsivity when methylphenidate is used in the treatment of children with ADHD and CD [7,14]. As well as positive effects on emotional dysregulation in this cohort of patients [8]. It was noted that studies have shown that the majority of medications show mixed results in treating CD, and with the stimulant medication methylphenidate, there is a need to use higher doses of methylphenidate up to 40mg/day to see a reduction in CD symptoms [9]. This matches our findings in this case, with the symptoms being markedly improved on a dose of 36mg/day. With use of methylphenidate, it is important to assess height, weight, cardiovascular assessment and blood pressure/pulse to monitor for adverse effects such as weight loss, hypertension and arrhythmias. NICE does not recommend an ECG before starting stimulants. Caution should be taken, and a cardiologist's opinion sought in cases of congenital heart disease, a first-degree relative with sudden cardiac death, shortness of breath compared to peers, palpitations, chest pain suspected of cardiac origin, a murmur on cardiac exam, signs of heart failure or blood pressure that would be classed as hypertension in adults [5]. Though no issues were noted in our case, it is important to be aware of these factors and potential adverse effects around use of methylphenidate. 

On its own, the early detection and treatment of ADHD are associated with improved outcomes, especially in cases like this that are early responders [10]. In this case, this is clearly highlighted due to the significant impact on education as well as family life. This was exacerbated due to the comorbidity with CD, risking the breakdown of the patient’s placement in his adoptive home. An important point to consider is that during the initial assessment, a large number of CD symptoms were identified and that not until further testing using the QB tests and Conners questionnaire the diagnosis of ADHD was confirmed. This highlights the risk of delay in diagnosis and appropriate management of ADHD and focus on management of CD would have potentially had a negative impact on this patient during a crucial period of his life, as he was completing high school.

## Conclusions

This comorbidity of ADHD with CD is a key factor in this case report. It was noted that after treatment of this patient's ADHD with methylphenidate, there was a significant reduction in aggressive and violent behaviour, resulting in a reduction in symptoms of CD. This could be attributed to the use of methylphenidate as well as other psychosocial interventions discussed in this report.

This case shows it is important to consider comorbid ADHD and CD in patients presenting with impulsive behaviours and aggression as their primary presenting complaints. Management with methylphenidate could have a therapeutic effect on these patients and their antisocial behaviours. Future research directions could focus on the early identification of ADHD in patients with CD. As seen with the patient presented earlier, treatment could lead to improvement in behaviours and family cohesion.
